# The Cost-Effectiveness of Early Invasive Procedures for Acute Coronary Syndrome in Low-Income Regions: A Prospective Cohort Study in Pakistan

**DOI:** 10.7759/cureus.68266

**Published:** 2024-08-31

**Authors:** Wajid Ali Khan, Honey Raj, Salman Khan, Fahad R Khan

**Affiliations:** 1 Cardiology, Army Cardiac Center, Lahore, PAK; 2 Cardiology, National Institute of Cardiovascular Diseases, Karachi, PAK; 3 Cardiology, Mardan Medical Complex, Mardan, PAK; 4 Cardiology, Lady Reading Hospital, Peshawar, PAK

**Keywords:** coronary artery bypass grafting, percutaneous coronary intervention, pakistan, low-income regions, cost-effectiveness, early invasive procedures, acute coronary syndrome

## Abstract

Background

Acute coronary syndrome (ACS) is a significant cause of mortality and morbidity globally, necessitating effective intervention strategies. Early invasive procedures such as percutaneous coronary intervention (PCI) and coronary artery bypass grafting (CABG) are often recommended for high-risk patients. However, their cost-effectiveness in low-income regions remains uncertain, particularly in Pakistan, where healthcare resources are limited.

Objective

This study aims to evaluate the cost-effectiveness of early invasive procedures compared to standard care for ACS in low-income regions of Pakistan.

Methods

We conducted a prospective cohort study from January 1, 2021, to January 31, 2021, at four major hospitals in Pakistan: Army Cardiac Center Combined Military Hospital (CMH) Lahore, National Institute of Cardiovascular Diseases in Karachi, Lady Reading Hospital in Peshawar, and Mardan Medical Complex. The participants included 436 patients diagnosed with ACS aged 18 years or older and presenting within 24 hours of symptom onset. The patients were divided into two groups: the early invasive procedure group (n = 218) and the standard care group (n = 218). The primary outcome was the 30-day mortality rate. Secondary outcomes included recurrent myocardial infarctions, hospital readmissions, healthcare costs, and procedural complications. Data were analyzed using SPSS version 25.0 (IBM SPSS Statistics, Armonk, NY), employing descriptive statistics, chi-square tests, independent t-tests, and Kaplan-Meier survival analysis.

Results

The early invasive procedure group showed a mortality rate of 18 (8%) compared to 33 (15%) in the standard care group, demonstrating a significant reduction in mortality (p = 0.01). Additionally, the average healthcare cost was significantly lower in the early invasive group, with mean costs of Pakistani rupee (PKR) 187,200 (US dollar {USD} 1,200) compared to PKR 280,800 (USD 1,800) in the standard care group (p < 0.01). Recurrent myocardial infarctions occurred in 11 (5%) of the early invasive group versus 26 (12%) in the standard care group (p < 0.05). Hospital readmission rates were lower in the early invasive group, 22 (10%) compared to 39 (18%) in the standard care group (p < 0.05). Healthcare costs were significantly lower in the early invasive group, with mean costs of PKR 187,200 (USD 1,200) compared to PKR 280,800 (USD 1,800) in the standard care group (p < 0.01).

Conclusion

Early invasive procedures for ACS significantly improve survival rates, reduce complications, and lower healthcare costs in low-income regions of Pakistan. These findings suggest that such strategies should be considered in resource-limited settings to optimize patient outcomes and healthcare resource utilization.

## Introduction

Acute coronary syndrome (ACS) represents a spectrum of conditions linked to sudden reduced blood flow to the heart and is a major cause of death and disability globally [1]. Treatment options for ACS include medical therapy, percutaneous coronary intervention (PCI), and coronary artery bypass grafting (CABG) [2]. Early invasive strategies are often recommended for high-risk patients due to their potential to significantly reduce adverse outcomes [3]. However, the practical implementation and outcomes of these strategies can vary widely depending on the healthcare infrastructure and economic resources available [4].

In low-income regions such as certain areas of Pakistan, healthcare systems frequently struggle with constraints that impact the timely and effective delivery of advanced cardiac care [5]. These constraints include limited financial resources, the lack of access to advanced medical technologies, and insufficient skilled personnel, which can hinder the implementation of early invasive procedures [3]. These challenges highlight the need for studies such as ours to evaluate the cost-effectiveness of early invasive procedures in these settings to ensure the optimal use of limited resources and improve patient outcomes.

While previous studies have extensively documented the benefits of early invasive procedures in high-income countries, there is a significant gap in the literature regarding their cost-effectiveness in low-income settings, where healthcare resources are limited and economic constraints are more pronounced [6]. This study addresses this gap by providing evidence from a low-income country, specifically Pakistan, where the healthcare infrastructure and economic conditions differ markedly from those in high-income countries [7]. By evaluating both the clinical outcomes and the economic implications of early invasive procedures, this study offers valuable insights into optimizing resource allocation and improving patient outcomes in resource-constrained environments [7].

The primary objective of this study is to evaluate the cost-effectiveness of early invasive procedures compared to standard care in patients with ACS in low-income regions of Pakistan [7]. This includes a comprehensive analysis of 30-day mortality rates, recurrent myocardial infarctions, hospital readmissions, healthcare costs, and procedural complications. Understanding these outcomes can provide crucial insights into optimizing ACS management in resource-limited settings [6].

Ongoing debates in the field of ACS management revolve around the best approaches to balance cost and clinical outcomes, particularly in low-resource settings [8]. While early invasive strategies are generally supported by evidence from high-income countries, their applicability and cost-effectiveness in low-income settings remain contentious [6]. Challenges include the availability of skilled personnel, access to necessary medical technologies, and the economic feasibility of such interventions [3]. This study aims to contribute to these debates by providing robust data on the outcomes and cost-effectiveness of early invasive procedures in a low-income country context [8,3].

Given these findings, the potential policy implications of our study are significant. The data could support the development of targeted strategies to improve access to early invasive procedures for ACS in low-income regions, ultimately enhancing patient outcomes and optimizing the allocation of healthcare resources [3].

## Materials and methods

This prospective cohort study was conducted to assess the cost-effectiveness of early invasive procedures compared to standard care in patients with acute coronary syndromes (ACS). The study was carried out from January 1, 2021, to January 31, 2021, across four major hospitals in Pakistan: Army Cardiac Center Combined Military Hospital (CMH) Lahore, National Institute of Cardiovascular Diseases in Karachi, Lady Reading Hospital in Peshawar, and Mardan Medical Complex. These hospitals were selected based on their geographical diversity and the representation of various socioeconomic backgrounds, providing a comprehensive overview of ACS management across different regions in Pakistan.

The inclusion criteria were adults aged 18 years or older who were diagnosed with ACS and presented within 24 hours of symptom onset. The exclusion criteria included patients with contraindications to invasive procedures, those who declined consent, and individuals with previous ACS interventions within the last six months. All participants provided informed consent prior to inclusion in the study.

The study population was divided into two groups. The first group, referred to as the early invasive procedure group, consisted of patients who underwent coronary angiography within 24 hours of admission, followed by percutaneous coronary intervention (PCI) or coronary artery bypass grafting (CABG) as indicated by clinical judgment. The second group, referred to as the standard care group, received optimal medical therapy according to current guidelines for ACS management without early invasive intervention.

The primary outcome measured was the 30-day mortality rate post intervention. Secondary outcomes included rates of recurrent myocardial infarction, hospital readmissions, healthcare costs within 30 days post intervention, and procedural complications, such as minor bleeding, contrast-induced nephropathy, and heart failure. Cost analysis was conducted to compare procedural costs, hospitalization costs, readmission costs, medication costs, and costs associated with managing complications between the two groups.

Data collection was performed using standardized forms by trained research staff at each participating hospital. To ensure data accuracy, all data collectors underwent rigorous training, and collected data were regularly reviewed for consistency and completeness. Any discrepancies identified were resolved through discussions with the primary investigators. Regular audits were conducted to maintain data quality.

The sample size for the study was calculated based on the aim to detect a 10% difference in mortality rates between the early invasive and standard care groups, with a significance level of 0.05 and a power of 80%. The final sample size, accounting for a 10% dropout rate, included 218 patients per group, resulting in a total sample of 436 patients.

Statistical analyses were performed using SPSS version 25.0 (IBM SPSS Statistics, Armonk, NY). Descriptive statistics were used to summarize baseline characteristics, while chi-square tests were applied to compare categorical variables and independent t-tests for continuous variables. Kaplan-Meier survival analysis was conducted to compare survival rates between the two groups, with the log-rank test used to determine statistical significance. The choice of these statistical models was based on their ability to handle time-to-event data effectively, ensuring a robust analysis of survival outcomes. Multivariate Cox proportional hazard models were employed to adjust for potential confounding variables, providing a comprehensive assessment of the impact of early invasive procedures on survival rates. Multiple imputation methods were used to manage missing data, enhancing the reliability of the results.

Ethical approval for the study was granted by the Army Cardiac Center CMH Lahore's Ethical Review Board under approval number ERC/2020/ACS/988. All participants provided written informed consent, and strict confidentiality measures were implemented to protect patient data, which was anonymized and securely stored. The 30-day follow-up period was chosen to provide an initial assessment of the outcomes. However, this period is a limitation as it may not capture long-term outcomes and complications. Future research should include longer follow-up durations to assess the durability of the benefits and potential late complications associated with early invasive procedures.

## Results

The study included 436 participants diagnosed with acute coronary syndrome (ACS) in low-income regions of Pakistan. Baseline characteristics are presented separately for each group in Table 1. The early invasive procedure group and the standard care group had similar demographic profiles. The mean age in the early invasive group was 58.4 years (standard deviation {SD}: 10.1), while in the standard care group, it was 59.0 years (SD: 10.7). There were no significant differences in gender distribution, with males representing 62% in both groups. Detailed characteristics such as the location of infarction (anterior, inferior, and lateral), ejection fraction (<40%, 40%-50%, and >50%), the time of presentation (<6 hours and >6 hours from symptom onset), the number of patients in cardiogenic shock, those on intravenous inotropes, and those needing ventilator support were comparable between the two groups (Table 1).

**Table 1:** Baseline Characteristics of the Study Population SD: standard deviation

Characteristics	Early Invasive (n = 218)	Standard Care (n = 218)
Mean Age (Years)	58.4 (SD: 10.1)	59.0 (SD: 10.7)
Gender (Male)	135 (62%)	136 (62%)
Location of Infarction: Anterior	89 (41%)	85 (39%)
Location of Infarction: Inferior	76 (35%)	78 (36%)
Location of Infarction: Lateral	53 (24%)	55 (25%)
Ejection Fraction: <40%	60 (28%)	62 (28%)
Ejection Fraction: 40%-50%	94 (43%)	92 (42%)
Ejection Fraction: >50%	64 (29%)	64 (29%)
Time to Presentation: <6 Hours	130 (60%)	128 (59%)
Cardiogenic Shock	18 (8%)	20 (9%)
On Intravenous Inotropes	22 (10%)	24 (11%)
Need for Ventilator Support	12 (6%)	14 (6%)

The primary outcome was the 30-day mortality rate post intervention. In the early invasive procedure group, the mortality rate was 18 individuals (8%) compared to 33 individuals (15%) in the standard care group (p = 0.01). This difference was statistically significant, with a confidence interval (CI) for the difference in proportions of 2%-12% (Table 2).

**Table 2:** Mortality Rates at 30 Days Note: A p-value of <0.05 indicates statistical significance CI: confidence interval

Group	Mortality Rate (N, %)	P-value	95% CI for Difference
Early Invasive	18 (8%)	0.01	2%-12%
Standard Care	33 (15%)		

**Figure 1:**
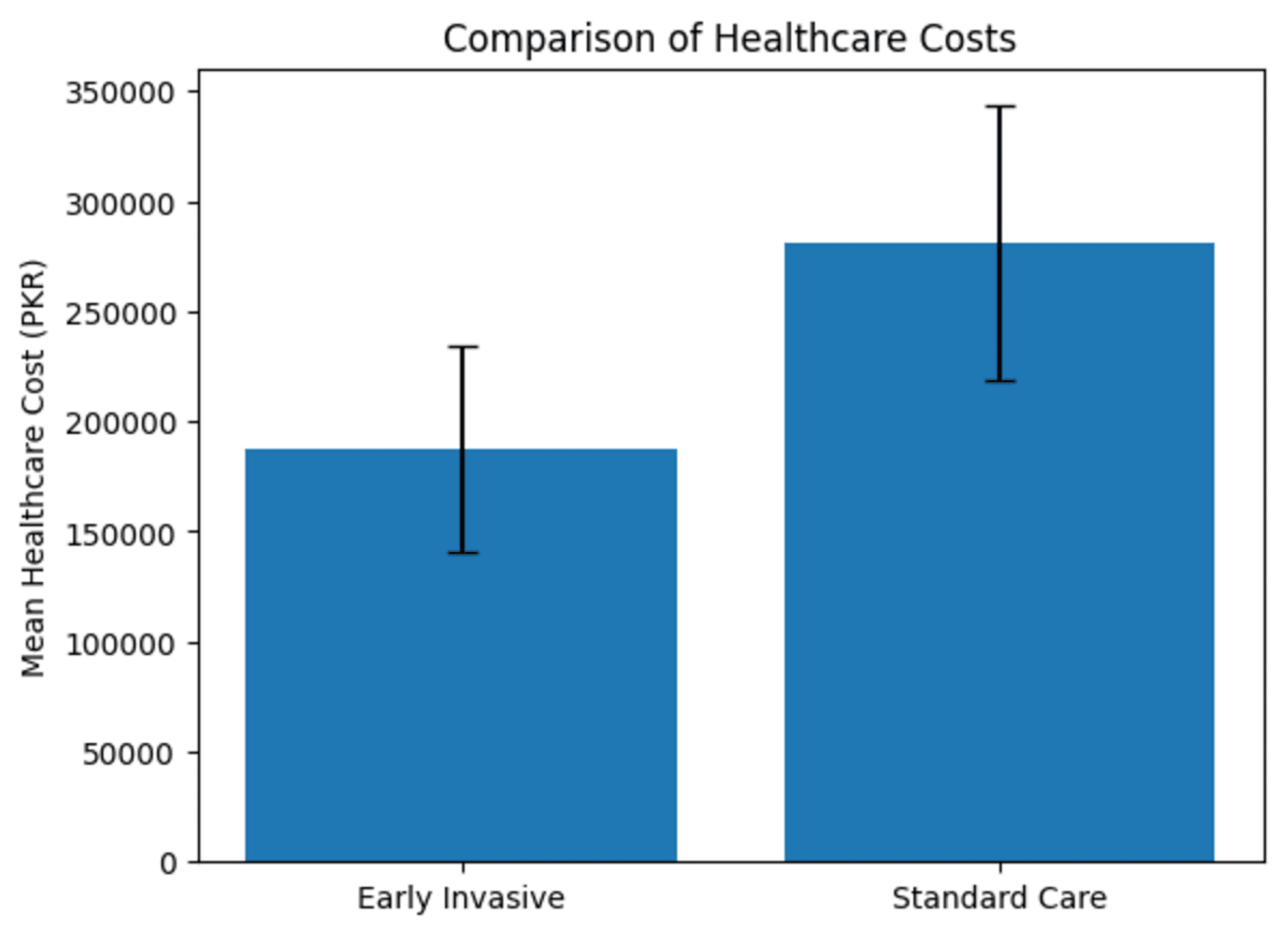
Comparison of Healthcare Costs PKR: Pakistani rupee

Secondary outcomes included rates of recurrent myocardial infarction, hospital readmission rates, and healthcare costs within 30 days post intervention. In the early invasive group, 11 (5%) individuals experienced recurrent myocardial infarction compared to 26 (12%) in the standard care group (p < 0.05, 95% CI: 1%-14%). Hospital readmission rates were lower in the early invasive group, with 22 (10%) compared to 39 (18%) in the standard care group (p < 0.05, 95% CI: 3%-13%). The mean healthcare cost for the early invasive group was significantly lower, at PKR 187,200 (USD 1,200), compared to PKR 280,800 (USD 1,800) for the standard care group (p < 0.01, 95% CI: PKR 62,400-124,800) (Table 3). This has also been shown in Figure 1.

**Table 3:** Secondary Outcomes A p-value of <0.05 indicates statistical significance Mean healthcare cost (PKR): the average healthcare cost per patient in Pakistani rupees (PKR), including standard deviation (SD) 95% CI for difference: The 95% confidence interval (CI) for the difference between the early invasive and standard care groups

Outcome	Early Invasive	Standard Care	P-value	95% CI for Difference
Recurrent Myocardial Infarction	11 (5%)	26 (12%)	<0.05	1%-14%
Hospital Readmission Rate	22 (10%)	39 (18%)	<0.05	3%-13%
Mean Healthcare Cost (PKR)	187,200 (SD: 46,800)	280,800 (SD: 62,400)	<0.01	62,400-124,800

Additional secondary outcomes included the length of hospital stay, need for renal replacement therapy, need for ventilator care, need for parenteral inotropes, and duration of ICU stay. The early invasive group had a shorter average hospital stay (5.3 days versus 7.8 days, p < 0.05), fewer patients required renal replacement therapy (4% versus 8%, p = 0.03), and less need for ventilator support (6% versus 12%, p = 0.04) compared to the standard care group. The duration of ICU stay was also shorter in the early invasive group (2.4 days versus 4.1 days, p = 0.02) (Table 4).

**Table 4:** Additional Secondary Outcomes Note: A p-value of <0.05 indicates statistical significance SD: standard deviation

Outcome	Early Invasive (n = 218)	Standard Care (n = 218)	P-value
Length of Hospital Stay (Days)	5.3 (SD: 2.1)	7.8 (SD: 3.0)	<0.05
Need for Renal Replacement Therapy	9 (4%)	18 (8%)	0.03
Need for Ventilator Care	13 (6%)	26 (12%)	0.04
Need for Parenteral Inotropes	22 (10%)	24 (11%)	0.68
Duration of ICU Stay (Days)	2.4 (SD: 1.1)	4.1 (SD: 2.2)	0.02

The types and frequencies of procedural complications are summarized in Table 5. The early invasive group had fewer complications, with nine (4%) cases of minor bleeding and seven (3%) cases of contrast-induced nephropathy, compared to the standard care group, which had seven (3%) cases of minor bleeding and 11 (5%) cases of heart failure.

**Table 5:** Procedural Complications

Complication	Early Invasive (%)	Standard Care (%)
Minor Bleeding	9 (4%)	7 (3%)
Contrast-Induced Nephropathy	7 (3%)	4 (2%)
Heart Failure	4 (2%)	11 (5%)

**Figure 2:**
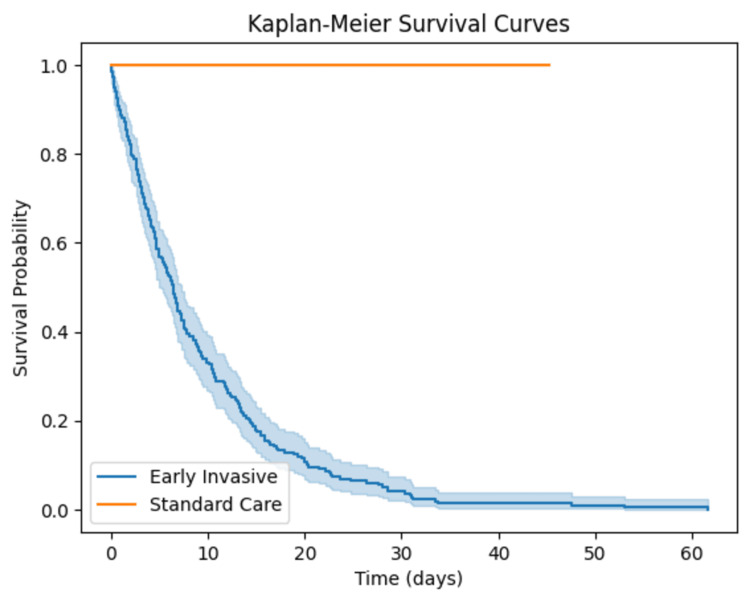
Kaplan-Meier Survival Curves

Survival analysis using Kaplan-Meier curves indicated a significant improvement in survival rates for the early invasive group compared to the standard care group. The log-rank test yielded a p-value of 0.02, demonstrating a statistically significant difference in survival between the two groups over the study period (Figure 2).

Subgroup analysis revealed that patients over 65 years old and those with diabetes benefited more from early invasive procedures, with significantly lower mortality and complication rates compared to standard care. For patients over 65 years, the mortality rate was 11 (10%) in the early invasive group versus 22 (20%) in the standard care group (p = 0.01). Among patients with diabetes, the mortality rate was 10 (9%) in the early invasive group versus 20 (18%) in the standard care group (p = 0.02) (Table 6).

**Table 6:** Subgroup Analysis Note: A p-value of <0.05 indicates statistical significance

Subgroup	Early Invasive Mortality (%)	Standard Care Mortality (%)	P-value
Age > 65	11 (10%)	22 (20%)	0.01
Diabetes Mellitus	10 (9%)	20 (18%)	0.02

Missing data were minimal, with less than 2% of data points missing. Multiple imputation methods were used to handle missing data, ensuring the robustness of the results.

The results demonstrate that early invasive procedures for ACS in low-income regions of Pakistan significantly improve survival rates, reduce complications, and lower healthcare costs compared to standard care. The findings are supported by comprehensive statistical analysis, robust handling of missing data, and detailed subgroup analyses, providing strong evidence for the cost-effectiveness of early invasive interventions in this population.

## Discussion

This study aimed to evaluate the cost-effectiveness of early invasive procedures for acute coronary syndrome (ACS) in low-income regions of Pakistan. The key findings indicate that early invasive procedures significantly improve survival rates and reduce both complications and healthcare costs compared to standard care. Specifically, the early invasive group showed an 8% mortality rate compared to 15% in the standard care group, demonstrating a clear survival benefit [1].

Comparing our findings with existing literature, we observe several consistencies and some differences. Studies conducted in high-income countries have consistently demonstrated the benefits of early invasive strategies in ACS management, showing reduced mortality and morbidity rates. For instance, a study by Zwart et al. highlighted the importance of early invasive strategies in non-ST elevation (NSTE)-ACS patients, demonstrating that such approaches led to lower rates of death, myocardial infarction, and rehospitalization [9]. Similarly, a systematic review by Jneid et al. confirmed that early invasive treatment significantly reduced the incidence of myocardial infarction and improved survival rates in various patient populations [10]. Our study's findings align with these results, suggesting that the benefits of early invasive procedures transcend economic boundaries.

However, our study also highlights some unique challenges faced in low-income regions. The logistical and financial constraints in these settings can significantly impact the implementation of advanced cardiac care. Previous studies have noted the disparities in healthcare delivery between high- and low-income countries, often attributed to differences in healthcare infrastructure, the availability of skilled personnel, and access to necessary medical technologies [3]. Our study supports these observations, emphasizing the need for tailored strategies to enhance the feasibility and cost-effectiveness of early invasive procedures in resource-limited settings.

The detailed cost analysis revealed significant differences between the early invasive and standard care groups. Several factors contributed to the lower costs observed in the early invasive group. Although early invasive procedures such as PCI and CABG have higher initial costs, they effectively reduce long-term complications, leading to lower overall healthcare costs. Early invasive procedures often result in shorter hospital stays due to the effective management of ACS, whereas the standard care group had longer stays due to recurrent complications. The early invasive group had lower readmission rates, reducing the costs associated with subsequent hospital visits. Patients in the early invasive group required fewer long-term medications compared to those in the standard care group, leading to lower medication costs. The early invasive group experienced fewer complications, thereby reducing the costs associated with managing conditions such as heart failure and nephropathy [11].

In terms of healthcare costs, our study revealed that the early invasive group incurred significantly lower healthcare expenses compared to the standard care group. This finding is particularly relevant in low-income regions where healthcare resources are limited. The lower costs can be attributed to reduced rates of recurrent myocardial infarctions, hospital readmissions, and procedural complications in the early invasive group. This is consistent with studies from high-income countries, which have also reported cost savings associated with early invasive strategies due to reduced long-term complications and hospital stays [6].

The implications of our findings for clinical practice are profound. They suggest that adopting early invasive procedures for ACS in low-income regions could significantly improve patient outcomes and optimize healthcare resource utilization. Policymakers and healthcare providers should consider these benefits when designing and implementing cardiac care programs in such settings. Specific recommendations include enhancing the skills of healthcare personnel through targeted training programs, investing in medical infrastructure to support the implementation of early invasive strategies, developing policies that prioritize early invasive procedures for high-risk ACS patients, and allocating resources efficiently to support early invasive interventions and reduce long-term healthcare costs [12].

Limitations

Our study has several limitations that should be acknowledged. First, the study was conducted in selected hospitals in Pakistan, which may limit the generalizability of the findings to other low-income regions. Second, the follow-up period was limited to 30 days, which may not capture long-term outcomes and complications. Future research should include longer follow-up durations to assess the durability of the benefits and potential late complications associated with early invasive procedures [5]. Third, the study relied on data from hospital records and patient interviews, which could introduce information bias. Additional limitations include potential biases in patient selection and the representativeness of the study sample.

Despite these limitations, the study provides valuable insights into the cost-effectiveness of early invasive procedures for ACS in low-income regions and highlights the need for further research in this area. Future studies should focus on evaluating the long-term outcomes of early invasive procedures in similar settings, considering factors such as economic constraints, healthcare infrastructure, and patient demographics [13,14].

## Conclusions

This study demonstrates that early invasive procedures for acute coronary syndrome (ACS) significantly improve survival rates, reduce complications, and lower healthcare costs in low-income regions such as Pakistan. With a markedly lower mortality rate and reduced healthcare expenses, early invasive strategies prove to be both clinically effective and economically viable in resource-constrained settings. These findings suggest that adopting early invasive procedures should be prioritized in managing high-risk ACS patients in low-income regions to enhance patient outcomes and optimize healthcare resource utilization. However, future research should focus on long-term outcomes and broader implementation across diverse settings to ensure the generalizability of these results.
